# 46例血液病患者肺孢子菌肺炎临床与影像特征

**DOI:** 10.3760/cma.j.issn.0253-2727.2023.02.006

**Published:** 2023-02

**Authors:** 慧明 伊, 春晖 徐, 栋林 杨, 青松 林, 英 李, 洪砚 孙, 四洲 冯

**Affiliations:** 中国医学科学院血液病医院（中国医学科学院血液学研究所），实验血液学国家重点实验室，国家血液系统疾病临床医学研究中心，细胞生态海河实验室，天津 300020 State Key Laboratory of Experimental Hematology, National Clinical Research Center for Blood Diseases, Haihe Laboratory of Cell Ecosystem, Institute of Hematology & Blood Diseases Hospital, Chinese Academy of Medical Sciences & Peking Union Medical College, Tianjin 300020, China

**Keywords:** 血液病, 肺孢子菌肺炎, 电子计算机断层扫描技术, Hematologic diseases, Pneumonia, pneumocystis, Tomography, spiral computed

## Abstract

**目的:**

总结血液病患者肺孢子菌肺炎（PJP）的初始肺CT影像特征。

**方法:**

回顾性分析2014年1月至2021年12月在中国医学科学院血液病医院诊断为PJP的46例血液病患者的临床及影像资料。所有患者均行多次胸部CT及相关实验室检查，依据初始肺CT表现进行影像分型，并分析不同临床特征分组下的不同影像分型分布。

**结果:**

46例患者中，男33例，女13例，中位年龄37.5（2～65）岁。肺泡灌洗液（BALF）六胺银染色确诊11例、临床诊断35例，临床诊断患者中16例经BALF宏基因组二代测序（BALF-mNGS）诊断、19例经外周血宏基因组二代测序（PB-mNGS）诊断。初始胸部CT表现分为4型，包括磨玻璃型25例（56.5％）、结节型10例（21.7％）、纤维化型4例（8.7％）、混合型5例（13.0％）。确诊、BALF-mNGS诊断和PB-mNGS诊断患者的CT类型构成比差异无统计学意义（*χ*^2^＝11.039，*P*＝0.087）。确诊患者和PB-mNGS诊断患者CT表现以磨玻璃型为主（63.6％、73.7％），BALF-mNGS诊断的患者CT表现以结节型为主，占37.5％。63.0％（29/46）的患者外周血淋巴细胞减少，25.6％（10/39）血清G试验阳性，77.1％（27/35）血清LDH水平升高。不同CT类型患者的外周血淋巴细胞减少率、G试验阳性率和LDH升高率的差异均无统计学意义（*P*值均>0.05）。

**结论:**

血液病患者PJP初始肺CT表现多样，以两肺多发磨玻璃影多见，结节型与纤维化型也是PJP初始CT表现。

## Abstract (en)

**Objective:**

To summarize the original CT features of Pneumocystis Jirovecii pneumonia in patients with hematological diseases.

**Methods:**

A retrospective analysis was carried out in 46 patients with proven pneumocystis pneumonia（PJP）in the Hospital of Hematology, Chinese Academy of Medical Sciences between January 2014 and December 2021. All patients had multiple chests CT and related laboratory examinations, imaging typing were conducted based on the initial CT presentation, and the distinct imaging types were analyzed against the clinical data.

**Results:**

In the analysis, there were 46 patients with proven pathogenesis, 33 males, and 13 females, with a median age of 37.5（2–65）years. The diagnosis was validated by bronchoalveolar lavage fluid（BALF）hexamine silver staining in 11 patients and clinically diagnosed in 35 cases. Of the 35 clinically diagnosed patients, 16 were diagnosed by alveolar lavage fluid macrogenomic sequencing（BALF-mNGS）and 19 by peripheral blood macrogenomic sequencing（PB-mNGS）. The initial chest CT presentation was categorized into 4 types, including ground glass（GGO）type in 25 cases（56.5％）, nodular type in 10 cases（21.7％）, fibrosis type in 4 cases（8.7％）, and mixed type in 5 cases（13.0％）. There was no substantial discrepancy in CT types among confirmed patients, BALF-mNGS diagnosed patients and PB-mNGS diagnosed patients（*χ*^2^＝11.039, *P*＝0.087）. The CT manifestations of confirmed patients and PB-mNGS diagnosed patients were primarily GGO type（67.6％, 73.7％）, while that of BALF-mNGS diagnosed patients were nodular type（37.5％）. Of the 46 patients, 63.0％（29/46）had lymphocytopenia in the peripheral blood, 25.6％（10/39）with positive serum G test, and 77.1％（27/35）with elevated serum lactate dehydrogenase（LDH）. There were no great discrepancies in the rates of lymphopenia in peripheral blood, positive G-test, and increased LDH among different CT types（all *P*>0.05）.

**Conclusion:**

The initial chest CT findings of PJP in patients with hematological diseases were relatively prevalent with multiple GGO in both lungs. Nodular and fibrosis types were also the initial imaging findings for PJP.

肺孢子菌肺炎（PJP）为恶性血液病患者常见的肺部感染性疾病，多发病急骤、病情严重，如得不到及时有效治疗病死率高（30％～59％），异基因造血干细胞移植（allo-HSCT）受者的病死率则更高，达48％～70％[1]。PJP临床主要表现为发热、呼吸困难等，但缺乏特异性指标。既往研究[2]–[4]显示PJP的影像学特征主要表现为双肺弥漫分布的磨玻璃影，具有一定特异性，有助于早期诊断和治疗。但其研究对象主要为非血液系统疾病患者，结论是否适用于血液病患者尚不明确。少数文献[1],[5]报道血液病患者合并PJP肺CT表现复杂多样。因此，本研究拟通过分析我院血液病患者合并PJP的胸部CT表现，探讨其初始CT特征，现报道如下。

## 病例与方法

1. 病例：回顾性分析我院2014年1月至2021年12月间诊断为PJP的患者临床及影像资料，其中病原学证据明确的患者46例，男33例、女13例，中位年龄37.5（2～65）岁。46例患者中allo-HSCT患者19例，原发病包括急性淋巴细胞白血病（ALL）8例、急性髓系白血病（AML）4例、骨髓增生异常综合征（MDS）4例、再生障碍性贫血（AA）2例、慢性髓性白血病（CML）1例；诊断为PJP的中位时间为+189（+135～+555）d。非移植患者27例，原发病包括非霍奇金淋巴瘤（NHL）9例、ALL 8例、AML 6例、AA 2例、多发性骨髓瘤（MM）1例、自身免疫性溶血性贫血（AIHA）1例。

所有患者均行胸部CT检查。46例患者中27例行支气管镜检查，肺泡灌洗液（BALF）行六胺银染色及宏基因组二代测序（mNGS），19例行外周血mNGS（PB-mNGS）检测。其他相关实验室检查包括血常规、G试验及LDH检测。

2. 诊断标准：参考已发表的PJP研究结果[6]–[7]制定诊断标准，本研究分为确诊和临床诊断，确诊标准：肺组织标本、BALF或痰液经六胺银染色，显微镜检出肺孢子菌包囊。临床诊断为未行六胺银染色或六胺银染色阴性，但需同时符合以下条件：①血液病患者经化疗或免疫抑制治疗后，或allo-HSCT后；②伴有呼吸困难、发热、干咳或胸闷等症状；③具有肺炎影像学表现；④BALF-mNGS或PB-mNGS检出肺孢子菌DNA片段；⑤抗PJP治疗有效。

3. 胸部CT检测：采用飞利浦Ingenuity 64排128层螺旋CT机，管电压120 kV，管电流200 mA，准直64×0.625 mm，矩阵512×512，扫描范围自肺尖至肺底，于深吸气末屏气扫描，层厚5 mm，间隔5 mm。肺窗窗宽1 000 Hu，窗位−700 Hu；纵隔窗窗宽400 Hu，窗位45 Hu。根据初始CT肺窗表现对所有患者进行归纳分型，共分为4种类型：①磨玻璃型：表现为两肺内中带弥漫分布的磨玻璃影，胸膜下区不受累（图1A）；②结节型：表现为肺内多发小结节影，包括间质结节、小叶中心结节及树芽征（图1B）；③纤维化型：表现为肺内多发索条影、支气管轻度牵拉扩张，部分可伴有肺实变（图1C）；④混合型：表现为多种影像征象并存，包括磨玻璃影、小结节及索条影共存（图1D）。

**图1:**
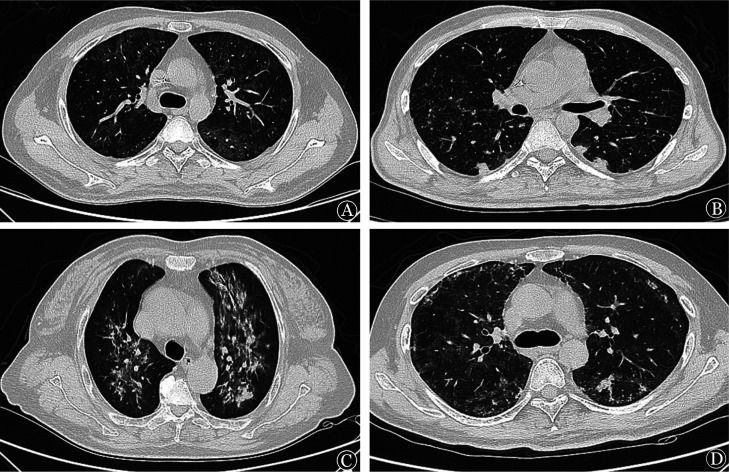
血液病患者肺孢子菌肺炎肺部CT的不同表现征象 A 磨玻璃型；B 结节型；C 纤维化型；D 混合型

4. 统计学处理：应用SPSS 22.0软件进行统计分析，分类资料以例数（构成比）进行统计描述，采用校正的卡方检验或Fisher精确概率法进行差异性分析，*P*<0.05为差异有统计学意义。

## 结果

1. 临床表现：46例患者中，发热37例（其中3例同时伴咳嗽、3例伴胸闷、3例伴呼吸困难），胸闷6例，咳嗽3例。

2. 病原体检测结果：27例接受支气管镜检查的患者中，11例BALF六胺银染色阳性，为确诊患者；16例BALF六胺银染色阴性但BALF-mNGS检出肺孢子菌DNA，为临床诊断患者。19例未行气管镜检查的患者经PB-mNGS检出肺孢子菌DNA，结合临床表现、影像特征、实验室检查及治疗转归综合诊断为PJP。8例患者BALF-mNGS同时检出多种病原体，其中3例检出CMV；16例患者PB-mNGS同时检出多种病原体，其中13例检出CMV。检测结果及治疗方案详见表1。mNGS检出CMV的患者，经PCR复核拷贝数均<1 000/ml。

**表1:** 35例临床诊断肺孢子菌肺炎（PJP）的血液病患者支气管镜肺泡灌洗液及外周血mNGS病原体检测结果及治疗方案

例号	标本来源	病原体	PJ序列数	抗感染方案
1	BALF	PJ	26 716	SMZ+MEM+LNZ
2	BALF	PJ+RV	321	SMZ+CAS
3	BALF	PJ+HHV7	3 086	SMZ+CAS
4	BALF	PJ	1 250	SMZ+PIS
5	BALF	PJ+CMV	20 980	SMZ+CAS+MP+PFD
6	BALF	PJ	12 496	SMZ+CAS+CSL+DEX
7	BALF	PJ	735	SMZ+CAS
8	BALF	PJ+TTV	412	SMZ+IPM+LNZ+VOR
9	BALF	PJ+CMV	50 916	SMZ+CAS+MFX+MP
10	BALF	PJ	360	SMZ+MP
11	BALF	PJ+EBV+CMV+TTV+PA	37	SMZ+CAS+CZA+LNZ+MEM+VOR
12	BALF	PJ	231	SMZ
13	BALF	PJ	119	SMZ+PIS+LNZ+VOR
14	BALF	PJ+PA	138	CAS+FEP
15	BALF	PJ+PY	5	SMZ+CAS+MEM+VAN
16	BALF	PJ	71 299	SMZ+CAS+MP
17	PB	PJ+CMV	1	SMZ
18	PB	PJ	18	SMZ+MEM+Posaconazole+AMB
19	PB	PJ+CMV	14	SMZ+CAS+MEM+LNZ
20	PB	PJ+CMV+Rhizopus	25	SMZ+CAS+Posaconazole+MFX+TGC
21	PB	PJ+CMV	12	CAS+LNZ+IPM
22	PB	PJ	20	CAS+MP
23	PB	PJ+CMV+HSV2	55	SMZ+Micafungin+CSL
24	PB	PJ+CMV	2	SMZ+CAS+MEM+LNZ+MP
25	PB	PJ+CMV	175	CAS+MEM+LNZ+VOR
26	PB	PJ+CMV	1	SMZ+CAS+MEM+Foscarnet
27	PB	PJ+CMV	7	SMZ+TGC+VOR+Foscarnet
28	PB	PJ	2	SMZ+CAS+MEM+Posaconazole
29	PB	PJ+CMV	1	CAS+CZA+TGC+VAN+Posaconazole
30	PB	PJ+TTV+HSV1+BKV	25	SMZ+CAS+MEM
31	PB	PJ+CMV+TTV	1	SMZ+CAS+MP
32	PB	PJ+HHV6	6	SMZ+VOR+MEM+CSL+VOR
33	PB	PJ+CMV	10	SMZ+CAS+MFX+VOR
34	PB	PJ+BKV+EBV+CMV	99	SMZ+CAS+MEM+VOR+AMB+GCV+LNZ
35	PB	PJ+BKV	249	SMZ+CAS+MP

**注** mNGS：宏基因组二代测序；BALF：肺泡灌洗液；PB：外周血；PJ：肺孢子菌；RV：鼻病毒；HHV：人类疱疹病毒；CMV：巨细胞病毒；TTV：细环病毒；EBV：EB病毒；PA：铜绿假单胞菌；PY：多瘤病毒；Rhizopus：根霉菌；HSV：单纯疱疹病毒；BKV：BK病毒；SMZ：复方磺胺甲唑；MEM：美罗培南；LNZ：利奈唑胺；CAS：卡泊芬净；PIS：哌拉西林他唑巴坦；MP：甲泼尼龙；PFD：吡非尼酮；CSL：头孢哌酮舒巴坦；DEX：地塞米松；IPM：亚胺培南；VOR：伏立康唑；CZA：头孢他啶阿维巴坦；FEP：头孢吡肟；VAN：万古霉素；Posaconazole：泊沙康唑；AMB：两性霉素B；MFX：莫西沙星；TGC：替加环素；Micafungin：米卡芬净；Foscarnet：磷甲酸；GCV：更昔洛韦

3. 实验室检查：46例患者全部行血常规检查，29例（63.0％）外周血淋巴细胞减少（<0.8×10^9^/L）；39例患者进行了G试验，10例（25.6％）G试验阳性（>95 ng/L）；35例患者检测了血清LDH水平，27例（77.1％）LDH增高（>248 U/L）；其中G试验和LDH双阳性者6例、双阴性者5例。46例患者全部行血浆白蛋白检测，其中30例（65.2％）白蛋白水平降低（<35 g/L）。

4. CT表现：46例患者初始胸部CT检查均可见异常，磨玻璃型26例（56.5％）、结节型10例（21.7％）、纤维化型4例（8.7％）、混合型6例（13.0％）。确诊、PB-mNGS诊断和BALF-mNGS诊断患者的CT类型分布见表2，差异无统计学意义（*χ*^2^＝11.039，*P*＝0.087）。确诊患者和PB-mNGS诊断患者均以磨玻璃型表现为主，分别占63.6％、73.7％，而BALF-mNGS诊断组CT表现以结节型居多，占37.5％，磨玻璃型占31.3％。19例allo-HSCT受者和27例非移植患者在CT类型差异亦无统计学意义（*χ*^2^＝5.847，*P*＝0.119），均以磨玻璃型表现为主，分别占57.9％、55.6％（表2）。不同CT类型患者的外周血淋巴细胞减少率、G试验阳性率和LDH升高率的差异均无统计学意义（表2）。

**表2:** PJP不同诊断依据、是否移植及实验室检查分组与CT表现征象分型的分布特征［例（％）］

分组	例数	磨玻璃型	结节型	纤维化型	混合型	*χ*^2^值	*P*值
诊断依据						11.039	0.087
确诊	11	7（63.6）	1（9.1）	2（18.2）	1（9.1）		
BALF-mNGS诊断	16	5（31.3）	6（37.5）	2（12.5）	3（18.8）		
PB-mNGS诊断	19	14（73.7）	3（15.8）	0（0.0）	2（10.5）		
是否移植						5.847	0.119
移植	19	11（57.9）	6（31.6）	0（0.0）	2（10.5）		
非移植	27	15（55.6）	4（14.8）	4（14.8）	4（14.8）		
是否淋巴细胞减少						1.985	0.575
是	29	15（51.7）	8（27.6）	2（6.9）	4（13.8）		
否	17	11（64.7）	2（11.8）	2（11.8）	2（11.8）		
G试验						6.168	0.104
阳性	10	8（80.0）	0（0.0）	1（10.0）	1（10.0）		
阴性	36	18（50.0）	10（27.8）	3（8.3）	5（13.9）		
是否LDH升高						4.677	0.197
是	27	18（66.7）	4（14.8）	3（11.1）	2（7.4）		
否	19	8（42.1）	6（31.6）	1（5.3）	4（21.1）		

**注** PJP：肺孢子菌肺炎；BALF：肺泡灌洗液；PB：外周血；mNGS：宏基因组二代测序；淋巴细胞减少：外周血淋巴细胞绝对计数<0.8×10^9^/L；G试验：（1,3）-β-D-葡聚糖试验，>95 ng/L定义为阳性；LDH升高：LDH>248 U/L

46例患者中12例伴有胸腔积液，其中11例为低蛋白血症；无胸腔积液的34例患者中19例为低蛋白血症，合并胸腔积液患者的低蛋白血症比例明显高于无胸腔积液者（91.7％对55.9％，Fisher，*P*＝0.035）。

5. 预后：46例患者中，1例患者病变持续进展，最终死于呼吸衰竭；2例患者放弃治疗（经电话随访确认死亡）；其余43例患者经抗PJP治疗后均完全康复。肺部CT显示病变大部分消失，仅少数遗留纤维索条影。

## 讨论

免疫抑制宿主合并PJP其CT多表现为肺门周围分布的磨玻璃影、胸膜下区一般不受累[8]，少数可表现为单发或多发结节、树芽征、实变、空洞、纵隔淋巴结增大和胸腔积液等[1],[9]–[12]。本组结果显示，血液病患者合并PJP最常见（56.5％）的CT表现为两肺弥漫多发磨玻璃影，与文献报道相符。但与既往研究不同的是，本组病例中21.7％的患者CT为表现为多发结节，这部分患者多数（60％）经BALF-mNGS检测获得病原学结果。此外另有13.0％和8.7％的患者CT表现为混合型和纤维化型，12例（26.1％）患者伴有胸腔积液。

既往研究指出PJP患者CT表现为肺小叶中心结节、边界不清的磨玻璃结节和实变影多为PJP合并CMV感染所致[13]；本组CT表现为结节型的患者中2例经PB-mNGS检出CMV-NDA，但经PCR复核拷贝数均<1 000/ml，抗PJP治疗后痊愈，因此我们认为结节型可能与CMV并不直接相关。李喆等[14]发现慢性肾病合并PJP患者CMV血症阳性率较高，但CMV并未加重肺部损伤和炎症反应，而是导致免疫系统损伤更重，我们的病例与之有相似之处。此外，有学者将结节型PJP称为肉芽肿型PJP，认为是由于抗PJP治疗不充分所致[15]；但本组10例均为初诊患者，后经抗PJP治疗痊愈。因此，结节型可能是血液病合并PJP初始肺CT的独特表现之一，需引起临床重视。牟向东等[16]认为纤维化是PJP晚期表现，但本组4例患者初诊即表现为纤维化型，且本组6例患者表现为混合型，经抗PJP治疗后肺内病灶均逐渐消散。因此，对于血液病合并PJP，纤维化型并不完全提示终末期改变，仍需积极治疗以争取良好结局。本组12例患者伴有胸腔积液，其中91.7％的患者存在低蛋白血症，这与既往研究[17]相符，即胸腔积液可能是低蛋白血症或者合并其他感染所致。

血液病患者合并PJP的CT表现复杂多样，对临床高度怀疑而肺部影像表现为非磨玻璃型的患者，不能轻易排除PJP的可能。应结合患者基础病、临床表现、实验室检查及CT表现综合诊断，有条件的患者需尽早进行病原学检测明确诊断。本组结果显示六胺银染色确诊患者与BALF-mNGS和（或）PB-mNGS检出病原体的患者相比，CT表现类型差异无统计学意义。提示对于非磨玻璃型CT表现的患者，多种方式可获得病原学结果，对于难以行支气管镜检查的患者，PB-mNGS检测对诊断也有重要的参考价值。

本组患者原发病包括ALL、AML、NHL、MDS、AA、CML、AIHA、MM，其中19例接受allo-HSCT、27例接受化疗或免疫抑制治疗，疾病谱和治疗方案与文献报道基本相符；Mansharamani等[18]研究显示PJP患者外周血淋巴细胞减少较为明显，尤以CD4^+^淋巴细胞减少为著，约91％的患者外周血CD4^+^淋巴细胞计数小于300/µl。由于本研究为回顾性分析，大部分患者在诊断为PJP时未行淋巴细胞亚群检测，结果显示63％的患者外周血淋巴细胞计数减少，以磨玻璃型和结节型为主；而外周血淋巴细胞计数正常的患者，以磨玻璃型为主。推测不同CT表现可能与淋巴细胞介导的免疫反应有一定关系[3]，即随着细胞免疫功能的减弱，肺部CT表现趋于多样化，提示对于具有罹患PJP高危因素而肺部CT表现为结节型、纤维化型和混合型的患者，不能完全除外PJP的诊断，需尽可能取得病原学证据。

此外，既往研究[19]–[20]显示G试验和LDH可作为PJP诊断的参考指标，但本组结果显示G试验阳性率仅为21.7％，明显低于上述文献报道；而LDH增高率为58.7％，与上述文献基本相符。研究认为G试验的阴性预测值可高达96％，低于80 ng/L几乎可以排除PJP[21]，但这些观点大部分是基于对非血液病患者的研究得出。我们的结果提示，血液病合并PJP的患者G试验阳性率偏低，一方面可能与入组病例选择偏倚有关，另一方面提示G实验对于血液病患者合并PJP的诊断价值有待于进一步研究，在临床工作中还需要重视LDH增高的诊断价值。其原因可能与血液病患者PJP菌量负荷较低有关[22]，也可能与检测试剂不同有关。然而，即使是采用80 ng/L作为cut-off值，本组也仅有16例（34.8％）患者G试验阳性。Mikulska等[23]对血液病患者G试验诊断效能进行荟萃分析发现，与其他患者群体比较恶性血液病患者合并PJP后G试验敏感性较低。Jiang等[24]针对国人非HIV免疫损伤患者合并PJP的研究显示，G试验的敏感度仅为67.4％，而mNGS的敏感度可达100.0％，且血液与BALF标本的mNGS检测一致性达100.0％。因此，mNGS对于血液病患者合并PJP的诊断具有重要的提示价值，尤其是不能行支气管镜检查的患者，PB-mNGS检测更突显其独特的临床价值，可能的机制是肺感染时孢子菌核酸入血能够被核酸测序识别[25]。但对于mNGS是否能够鉴别PJP定植和感染尚无确切结论，有待于深入研究。

综上，本研究提示G试验阳性、LDH增高、肺CT以磨玻璃型表现为主对血液病患者合并PJP的诊断有重要提示价值。但本组病例数目相对较少，结果和结论可能存在偏倚，有待于大样本研究进一步探索。
